# Hydrogen Sulfide as a Toxic Product in the Small–Large Intestine Axis and its Role in IBD Development

**DOI:** 10.3390/jcm8071054

**Published:** 2019-07-19

**Authors:** Ivan Kushkevych, Dani Dordević, Peter Kollar, Monika Vítězová, Lorenzo Drago

**Affiliations:** 1 Department of Experimental Biology, Faculty of Science, Masaryk University, Kamenice 753/5, 62500 Brno, Czech Republic; 2 Department of Plant Origin Foodstuffs Hygiene and Technology, Faculty of Veterinary Hygiene and Ecology, University of Veterinary and Pharmaceutical Sciences, 61242 Brno, Czech Republic; 3 Department of Human Pharmacology and Toxicology, Faculty of Pharmacy, University of Veterinary and Pharmaceutical Sciences, 61242 Brno, Czech Republic; 4 Department of Biomedical Sciences for Health, University of Milan, 20122 Milan, Italy

**Keywords:** small–large intestine axis, hydrogen sulfide, *Desulfovibrio*, bowel disease, colitis

## Abstract

The small–large intestine axis in hydrogen sulfide accumulation and testing of sulfate and lactate in the gut–gut axis of the intestinal environment has not been well described. Sulfate reducing bacteria (SRB) of the *Desulfovibrio* genus reduce sulfate to hydrogen sulfide and can be involved in ulcerative colitis development. The background of the research was to find correlations between hydrogen sulfide production under the effect of an electron acceptor (sulfate) and donor (lactate) at different concentrations and *Desulfovibrio piger* Vib-7 growth, as well as their dissimilatory sulfate reduction in the intestinal small–large intestinal environment. Methods: Microbiological, biochemical, and biophysical methods, and statistical processing of the results (principal component and cross-correlation analyses) were used. Results: *D. piger* Vib-7 showed increased intensity of bacterial growth and hydrogen sulfide production under the following concentrations of sulfate and lactate: 17.4 mM and 35.6 mM, respectively. The study showed in what kind of intestinal environment *D. piger* Vib-7 grows at the highest level and produces the highest amount of hydrogen sulfide. Conclusions: The optimum intestinal environment of *D. piger* Vib-7 can serve as a good indicator of the occurrence of inflammatory bowel diseases; meaning that these findings can be broadly used in medicine practice dealing with the monitoring and diagnosis of intestinal ailments.

## 1. Introduction

The destination of food remains from the small intestine, together with microbial biomass, is the large intestine, which represents an open system of the small–large intestine axis [1]. This means that the large intestine is a reactor for constant microorganism cultivation [2]. This fact is supported by the calculation that 200 g of digestive material is present in the large intestine of an adult human [2,3]. The intestinal lumen biomass includes almost 55% microorganisms, which are present in the total fecal content [1,4,5]. The microbial mass in the large intestine is 10^11^–10^12^ cells/g feces of the following dominant genera: *Bifidobacterium*, *Bacteroides*, *Lactobacillus*, *Escherichia*, *Enterococcus*, *Atopobium*, *Faecalibacterium*, *Clostridium*, and 40 other bacterial species that represent 99% of the colon microbiota [1,4,6,7]. 

The majority of these bacteria are able to cleave complex organic compounds in the fermentation process and they produce molecular hydrogen, different acids including acetate and lactate, and other compounds. The production of lactate depends on the fermentative properties of lactic acid bacteria (e.g., *Bifidobacterium*, *Lactobacillus*, and *Streptococcus*) [4]. This means that lactate and acetate can be also used by other groups of microorganisms. These compounds can be used as electron donors and carbon sources in the metabolic processes of microorganisms [7,8,9,10]. Intestinal microbiota is especially involved in the digestion processes of short-chain fatty acids [4]. The physiology and metabolism of humans is highly dependent on intestinal microorganisms and consequently affects human physiological functions and health [1,2,3,11,12]. On the other hand, another important component of human physiological status is the capability of the intestinal system to absorb sulfate for amino acid development, such as cysteine and methionine, and its regular involvement in assimilation processes. Concentrations of sulfate in the intestine are dependent on human diet since sulfate is present differently in different food commodities [13,14,15,16]; another factor is that sulfate absorption is done individually in each human, meaning that the total sulfate content in the intestine is highly influenced by eating habits. The importance of daily sulfate intake can be emphasized by the fact that staple food commodities (such as some breads) represent high sulfate sources (>10 µmol/g) as do popular beverages such as beers and wines (>2.5 µmol/g) [13].

The remnants of sulfate that are not absorbed by the intestines and the presence of lactate make a good environment for sulfate-reducing bacteria (SRB) that are regularly found in human and animal intestines [1,4,17,18,19,20,21]. SRB use sulfate as the final electron acceptor in the process of dissimilatory sulfate reduction and form the end product of hydrogen sulfide [22,23,24,25,26,27]. Different organic compounds, including lactate, can be exogenic electron donors for this process and can be oxidized to acetate [18,28]. *Desulfovibrio* genus is the dominant SRB in the human intestine [5,22]. Previous studies indicated a correlation between the SRB intestinal presence and ailments, such as cholecystitis, brain abscesses, and abdominal cavity ulcerative enterocolitis, making *Desulfovibrio* species an important factor during both mono- and poly-microbial infections of the gastrointestinal tract [2,3,4,12]. Consequently, the prevalence of SRB in the intestines is dependent on the occurrence of sulfate and lactate presence in the gut. It is also important to stress that the intestinal environment should be monitored due to its influence on SRB since a connection with these bacterial strains and inflammatory bowel diseases (IBD) has been found [1,2,3,11]. The effects of sulfate and lactate at different concentrations on intestinal *Desulfovibrio* species growth and their sulfate reduction parameters have not been well studied. 

The aim of this research was to find correlations between different sulfate and lactate concentrations and *Desulfovibrio piger* Vib-7 growth parameters and their dissimilatory sulfate reduction in the small–large intestinal environment.

## 2. Experimental Section

### 2.1. Bacterial Culture and Cultivation

The sulfate-reducing bacteria of the *Desulfovibrio piger* strain Vib-7 was used as the object of the study. This strain was isolated from the human large intestine and identified based on physiological and biochemical properties and sequence analysis of the 16S rRNA gene. The accession number in GenBank is KT881309.1. The strain of SRB was kept in the collection of microorganisms at the Laboratory of Anaerobic Microorganisms of the Department of Experimental Biology at Masaryk University (Brno, Czech Republic).

The bacterial culture was grown in modified liquid Postgate C medium [23] for 72 h at 37 °C under anaerobic conditions [29]. The following sodium sulfate concentrations were prepared in medium: 0.87 mM, 1.75 mM, 3.5 mM, 7 mM, 10.5 mM, and 17.5 mM. Different concentrations of electron donors and their effect in the medium were determined in the presence of sodium lactate (4.45 mM, 8.9 mM, 17.8 mM, 35.6 mM, 53.4 mM, or 89 mM). The control medium consisted of 3.5 mM sulfate and 17.8 mM lactate. The determination of biomass, sulfate, hydrogen sulfide, lactate, and acetate concentrations were determined after 12, 24, 36, 48, 60, and 72 h.

### 2.2. Bacterial Biomass Determination

In total, 1 mL of liquid medium without Mohrʼs salt in a plastic cuvette was measured in a biophotometer (Eppendorf BioPhotometer®D30, Hamburg, Germany) as a blank. The same procedure with the bacterial suspension was performed. The optical density (OD_340_) was always measured before the experiments to provide approximately the same amount of SRB in each experiment [7].

### 2.3. Sulfate Determination

The sulfate concentration in the liquid medium was measured by turbidimetric method after 12 h intervals of cultivation. In total, 40 mg/L BaCl_2_ solution was prepared in 0.12 M HCl and mixed with glycerol in a 1:1 ratio. The supernatant of the sample was obtained by centrifugation at 5000× *g* at 23 °C and 1 mL was added to 10 mL of BaCl_2_:glycerol solution and carefully mixed. The absorbance of the mixed solution was measured after 10 min at 520 nm (Spectrosonic Genesis 5, Ecublens, Switzerland). A cultivation medium without bacteria growth was used as a control [30].

### 2.4. Hydrogen Sulfide Determination

The concentration of hydrogen sulfide was determined in cultivation medium after different time intervals. In total, 1 mL of the sample was added to 10 mL of a 5 g/L solution of zinc acetate and 2 mL of 0.75 g/mL p-aminodimethylaniline in a solution of sulfuric acid (2 M). The mixture stood for 5 min at room temperature. After that, 0.5 mL of 12 g/L solution of ferric chloride dissolved in 15 mM sulfuric acid was added. After standing another 5 min at room temperature, the mixture was centrifuged 5000× *g* at 23 °C. The absorbance of the mixture was determined at a wavelength of 665 nm by a spectrophotometer (Cecil Aquarius CE 7200 Double Beam Spectrophotometer, London, UK) [31,32].

### 2.5. Lactate and Acetate Determination

The measurement was repeated in the same manner using a cultivation medium and it served as the control sample. Measurements of lactate concentration using a lactate assay kit (Sigma-Aldrich, Catalog Number MAK064, Prague, Czech Republic) were carried out. Accumulation of acetate ions in the process of bacterial growth in the medium was determined using the acetate assay kit (Abnova, Colorimetric, Catalog Number KA3764, Prague, Czech Republic).

### 2.6. Statistical Analysis

Using the experimental data, the basic statistical parameters (M—mean, m—standard error, M ± m) were calculated. The accurate approximation was when *p* ≤ 0.0533 [33]. Statistical significance was measured with the use of principal component analysis (PCA) that gave overall differences among compared groups. Statistical analysis was done by SPSS 20 statistical software (IBM Corporation, Armonk, NY, USA). Plots were built by software package Origin7.0 (Northampton, UK).

## 3. Results

**Figure 1:**
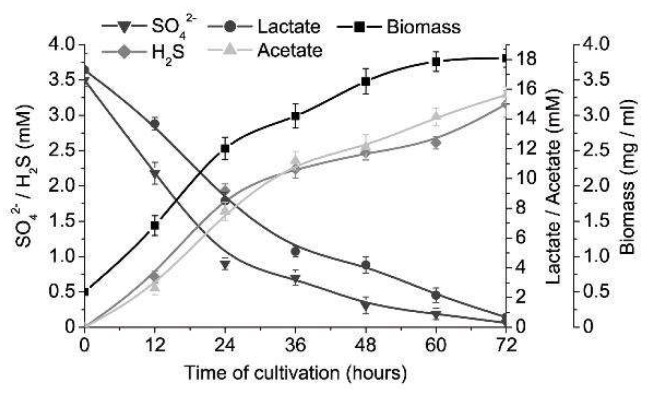
The growth of *D. piger* Vib-7 and their sulfate reduction.

Intestinal sulfate-reducing bacteria, *D. piger* Vib-7, showed the highest rate (biomass accumulation, sulfate and lactate consumption, and sulfide and acetate production), both increasing and decreasing trends, until the 60th h of cultivation in the control (3.5 mM of sulfate and 17.3 mM of lactate) medium (Figure 1). The stationary growth phase was achieved after 60 h of cultivation and the following percentage decreases and increases in contents were measured: biomass (increased by 87%), sulfate (decreased by 95%), sulfide (increased by 83%), lactate (decreased by 88%) and acetate (increased by 91%). Relative growth and survival of *D. piger* Vib-7 achieved the highest percentages at 7 mM of sulfate and 35.6 mM of lactate. Higher concentrations than these resulted in the stability of relative growth and it stayed at the same level during 12 to 48 h. Lower concentrations of sulfate (<3.5 mM) and lactate (<17.8 mM) were not enough for the achievement of maximum growth parameters.

**Figure 2:**
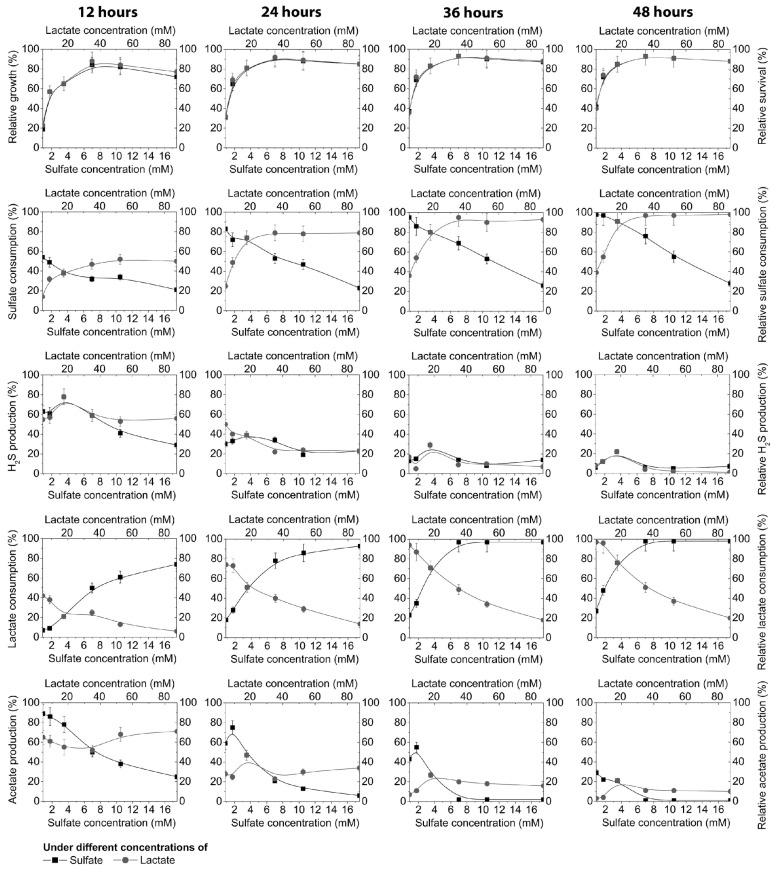
Growth of *D. piger* Vib-7, and their survival and sulfate reduction parameters during 12, 24, 36, and 48 h of cultivation: the effect of electron acceptor (sulfate)/donor (lactate) at different concentrations (columns: first = 12 h, second = 24 h, third = 36 h, fourth = 48 h).

As can be seen in Figure 2, the consumption of sulfate was highly dependent on its different concentrations in cultivation medium, time of cultivation, and the presence of lactate donors (it was constant at 17.8 mM of lactate). 

After 12 h, 54% of the sulfate was consumed in medium with lower sulfate concentrations (0.87 mM), although after 48 h, sulfate was almost consumed (98%) at the lowest concentration (0.87 mM) and only 28% at the highest sulfate concentration, where 72% was not used during this time period. Under other conditions, the following changes occurred: different lactate concentrations (4.45 mM, 8.9 mM, 17.8 mM, 35.6 mM, 53.4 mM, or 89 mM) were added in the cultivation medium and the consumption of the sulfate was measured.

As can be seen in Figure 2, sulfate consumption depended not only on its concentration, but was also strongly correlated with the concentration of an electron donor (lactate). Within this environment 14% of the sulfate was used at the lowest lactate concentration (4.45 mM) and 50% at 89 mM of lactate in the medium after 12 h of cultivation. The time of cultivation and lactate concentration increased the sulfate reduction in the medium. After 48 h, sulfate was used only 39–55% at the lowest concentrations of lactate (4.45–8.9 mM) because not enough electron donor was present. However, increasing the lactate concentrations from 35.6 to 89 mM induced 91–98% consumption of sulfate. The same trend was noticed with the lactate consumption. It could be seen that the production of sulfide was not very much influenced by the concentration of electron acceptor (0.87 mM to 17.5 mM), or the electron donor (4.45 mM to 89 mM), in the time interval from 24 to 48 h. The hydrogen sulfide production during this time period was stable. The highest production (78%) of sulfide was accumulated during the first 12 h and gradually decreased to 39%, 29%, and 22%, after 24, 36, and 48 h, respectively, under the conditions of 3.5 mM sulfate and 17.8 mM lactate. A similar trend was noticed in acetate production, although acetate production was more influenced by the sulfate and lactate concentration in the medium, as well as by the cultivation time. The highest production of acetate was until the 36th h of cultivation and after this period it decreased (Figure 2).

**Figure 3:**
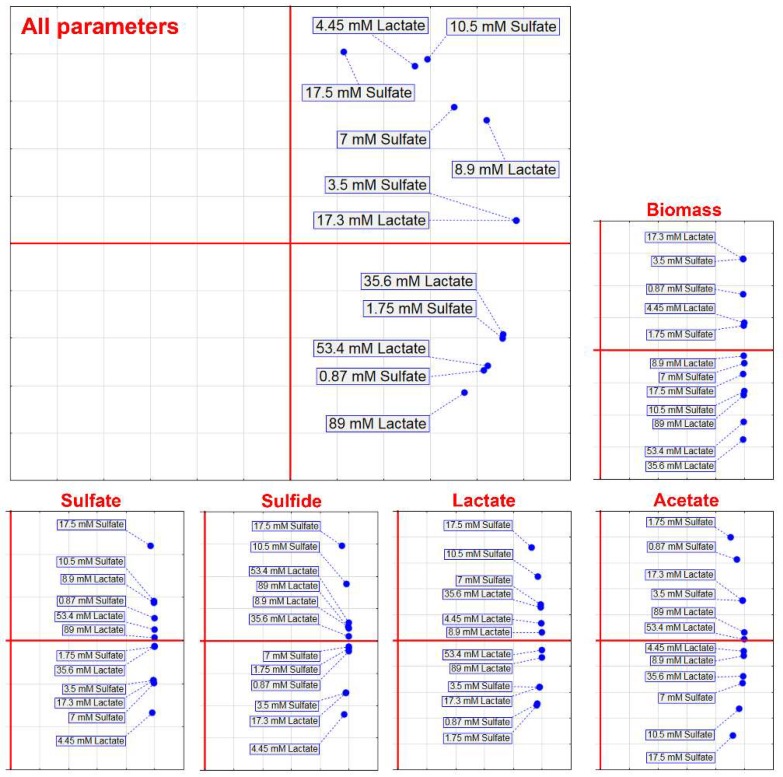
Principal component analysis of the *D. piger* Vib-7 growth and the parameters of sulfate reduction under the effect of electron acceptor (sulfate)/donor (lactate) at different concentrations.

Based on different concentrations of electron acceptor and donor, PCA was carried out (Figure 3) that included the separate parameters of biomass, sulfate and lactate consumption, and H_2_S and acetate production, as well as PCA that included all mentioned parameters. 

PCA that included separate parameters did not show clusters that would indicate a trend observed in Figure 2, but PCA that included all parameters showed that concentrations of 53 mM lactate and 0.87 mM sulfate, 1.75 mM sulfate and 35.6 mM lactate, and 3.5 mM sulfate and 17.3 mM lactate formed separated clusters. These findings indicated that lower concentrations of sulfate were prevailing in an environment with higher concentrations of lactate.

**Figure 4:**
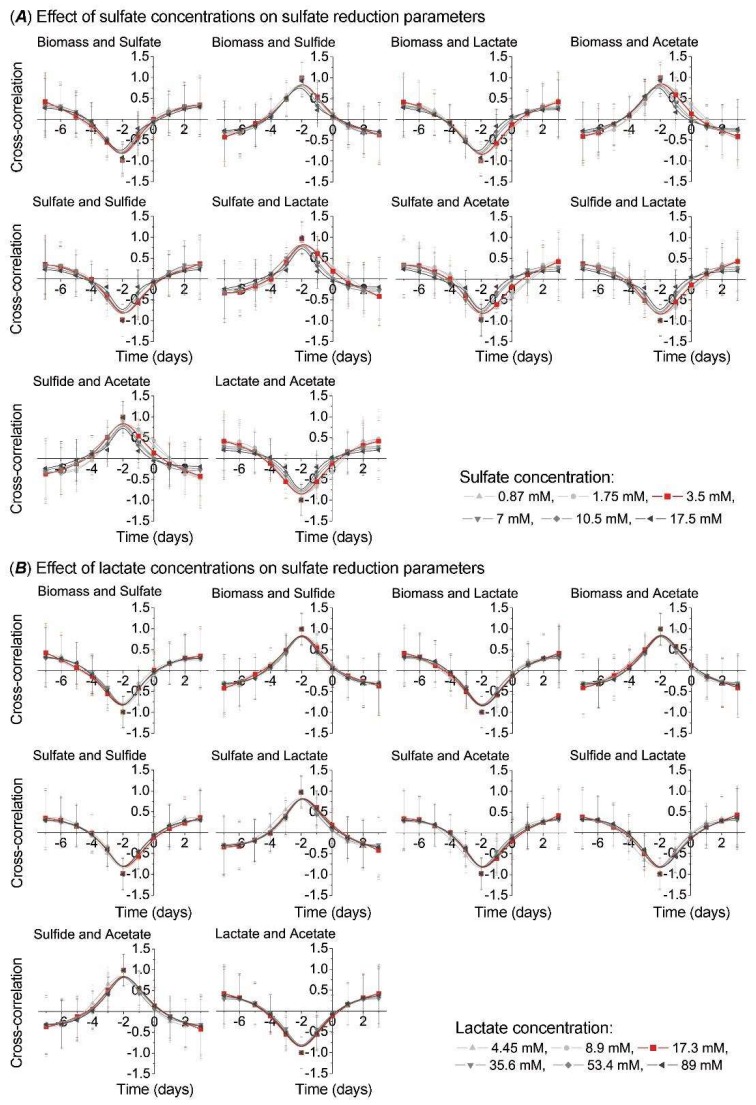
Cross-correlation analysis between growth (biomass) and sulfate reduction parameters under the effect of electron acceptor (sulfate)/donor (lactate) at different concentrations.

To observe side shifts in the process of sulfate reduction in the intestinal environment, including different concentrations of sulfate and lactate, cross correlation analysis was carried out between the following parameters: biomass and sulfate, biomass and sulfide, biomass and lactate, biomass and acetate, sulfate and sulfide, sulfate and lactate, sulfate and acetate, sulfide and lactate, sulfide and acetate, and lactate and acetate (Figure 4). 

The higher sulfate concentrations resulted in a shift to the left or right side on the Y axis, in comparison to the control sample (red line in Figure 4A), by all parameters, although more significantly by the following parameters: biomass and lactate, biomass and acetate, sulfate and lactate, and sulfate and acetate. Oppositely, lactate concentration effect did not cause similar shifting on the Y axis (Figure 4B).

**Figure 5:**
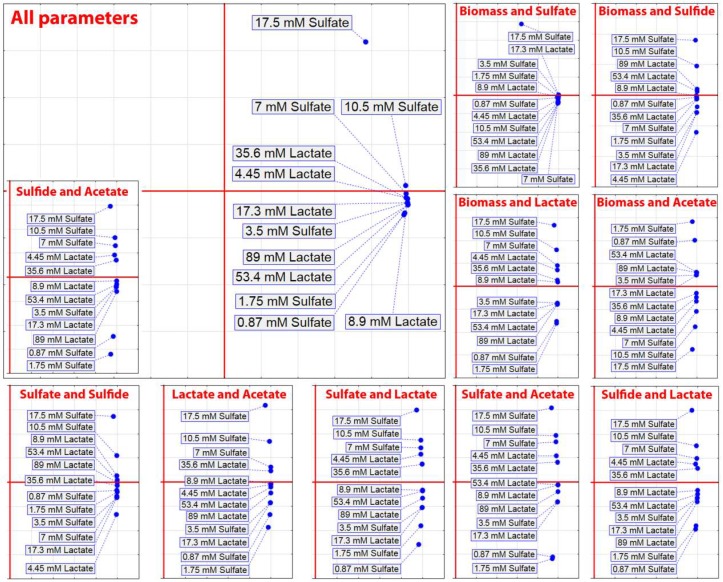
Principal component analysis of the *D. piger* Vib-7 growth and the parameters of sulfate reduction based on cross-correlation analysis.

PCA of the *D. piger* Vib-7 growth and the parameters of sulfate reduction based on cross-correlation analysis clearly showed an isolated cluster of the highest sulfate consumption (17.5 mM) in comparison with other concentrations. This means that bacteria were not able to fully consume these high sulfate concentrations during 48 h of cultivation (Figure 5). 

The kinetic parameters of *D. piger* Vib-7 growth under the effect of electron acceptor (sulfate)/donor (lactate) at different concentrations are shown in Table 1. 

Under sulfate concentrations of 10.5 mM the shortest lag phase was measured and specific maximum rate of growth (µmax) was the fastest at 7.0 mM of sulfate. Under electron donor (lactate) concentrations the shortest lag phase and the fastest specific maximum rate of growth were detected at 53.4 mM and 35.6 mM of lactate, respectively.

## 4. Discussion

The sulfate consumption and sulfide production, and the lactate consumption and acetate accumulation are important factors influencing the intestinal environment [7,8,9,10]. Intestinal sulfate-reducing bacteria, especially *Desulfovibrio* genus, are often found in the intestines and feces of people and animals with IBD. One of the main roles in the development of colitis, among other factors, can also be the species of this genus. These bacteria use sulfate as a terminal electron acceptor and organic compounds as electron donors in their metabolism [6,7]. This fact leads us to the conclusion that sulfate present in the daily diet plays an important role in the development of bowel disease. Sulfate is present mainly in the following food commodities: some breads, soya flour, dried fruits, brassicas, and sausages, as well as some beers, ciders, and wines. These data indicate that sulfate intake is highly dependent on diet and the small–large intestine axis [13].

In our previous research, principal component analysis indicated that the *Desulfovibrio* strains from individuals with colitis were grouped in one cluster by biomass accumulation and sulfide production, and the strains from healthy individuals formed another cluster by the same parameters. Sulfate and lactate consumption measured over time showed a negative correlation (Pearson correlations, *p* < 0.01). The linear regression (*R*^2^) was lower in biomass accumulation and hydrogen sulfide production. Thus, biomass accumulation and sulfide production, together with measured kinetic parameters, play an important factor in bowel inflammation, including ulcerative colitis. Additionally, acetate produced by SRB can also be in synergic interaction with H_2_S, while sulfate consumption and lactate oxidation likely represent minor factors in bowel disease [16].

Our results provide an opportunity to find the optimum growing point of the bacteria. The study confirmed an intense growth of *D. piger* Vib-7 in the presence of higher concentrations of electron acceptor and donor, though the consequence is an intensive accumulation of sulfide and acetate. Data from the literature indicate that these conditions can be the cause of ulcerative colitis that can lead to cancer of the bowel. This statement is supported by the fact that hydrogen sulfide negatively affects intestinal mucosa and epithelial cells, inhibits the growth of colonocytes [4,14,15,16,17,18,34,35,36,37], causes phagocytosis, causes the death of intestinal bacteria [4,12,24], and induces hyperproliferation and metabolic abnormalities of epithelial cells [12]. The high level of metabolites and the presence of SRB are connected with the inflammation of the colon [4,6,36]. Therefore, the integrity of colonocytes is maintained by hydrogen sulfide concentration [35,36,37]. Sulfide production is higher among SRB isolated from individuals with ulcerative colitis [5,6].

Other research describing cross-correlation parameters of the SRB metabolic process indicated that the strains isolated from people with colitis shifted to the right side of the Y axis by biomass accumulation, sulfate consumption, lactate oxidation, as well as hydrogen sulfide and acetate production, compared with the strains isolated from healthy individuals. Different percentages were observed in shifting to the right side of the Y axis: biomass accumulation 26%, sulfate consumption 1.5%, and sulfide production 5% [14]. It should be noted that the intestinal microbiota is a very complex system that may limit this study. There are a lot of interactions with clostridia, methanogens, lactic acid bacteria, etc. However, a central role in the development of IBD, especially ulcerative colitis, is SRB [1,2,3,11]. This bacterial group, producing hydrogen sulfide, can inhibit other microbiota, including lactic acid bacteria, methanogens, and many other intestinal microorganisms [2].

A diet high in sulfate ions (preservatives added to food often contain sulfur oxides) causes an increase in hydrogen sulfide concentration by SRB in rumens. The studies have revealed that the western diet contains over 16.6 mmol sulfate/day [13] and the feces of approximately 50% of healthy individuals contain SRB (up to 92% belong to the genus *Desulfovibrio*) [1,5]. Sulfate polysaccharides such as mucin, chondroitin sulfate, and carrageenan are broadly consumed, and they represent good sources of sulfate for SRB [24]. It should also be noted that hydrogen sulfide can be toxic not only for intestinal cells, but also for its producers. The highest toxicity of H_2_S was measured in the presence of concentrations higher than 6 mM, where growth was stopped, though metabolic activities were not 100% inhibited. These findings are confirmed by cross correlation and principal component analysis that clearly support the above mentioned results. The presence of 5 mM H_2_S resulted in a two times longer lag phase and generation time was eight times longer. The results confirmed toxicity of H_2_S toward *Desulfovibrio* [18]. Beside sulfate and lactate, terminal oxidative processes in the human large intestine could be involved in the activities of SRB, and consequently the production of hydrogen sulfide in high concentrations that can cause inflammatory bowel disease development.

## 5. Conclusions

The study gave more information about the intestinal environment in vitro concerning sulfate and lactate concentrations and their effects on the growth parameters of *Desulfovibrio piger* Vib-7. Almost total consumption of sulfate and lactate was achieved after 60 h of cultivation, though the best relative growth and stability was measured at 7 mM and 35.6 mM of sulfate and lactate, respectively. PCA including separated parameters did not show combined clusters, but PCA based on all parameters showed that different concentrations of sulfate and lactate formed separated clusters. These obtained results represent the main findings of the research, indicating that SRB would grow at the highest level under these experimentally simulated conditions. These conditions are an indicator of higher SRB activity that can lead to the development of IBD, and further studies will certainly focus more on the intestinal environment concerning SRB not only in vitro, but also in vivo.

## Figures and Tables

**Table 1:** Kinetics of *D. piger* Vib-7 growth under the effect of electron acceptor/donor.

Electron Acceptor (Sulfate)				Electron Donor (Lactate)			
Sulfate (mM)	Lag-Phase (h)	Generation Time T_d_ (h)	µ_max_ (h^−1^)	Lactate (mM)	Lag-Phase (h)	Generation Time T_d_ (h)	µ_max_ (h^−1^)
**0.87**	38.2 ± 3.5	16.5 ± 1.5	0.009 ± 0.0001	**4.45**	36.6 ± 3.7	14.5 ± 1.35	0.009 ± 0.008
**1.75**	5.9 ± 0.46	4.3 ± 0.44	0.02 ± 0.001	**8.9**	7.1 ± 0.66	3.6 ± 0.33	0.03 ± 0.001
**3.5**	6.4 ± 0.62	1.8 ± 0.15	0.05 ± 0.004	**17.3**	6.4 ± 0.60	1.8 ± 0.12	0.05 ± 0.004
**7.0**	7.4 ± 0.73	1.1 ± 0.10	0.08 ± 0.007	**35.6**	4.9 ± 0.43	1.1 ± 0.10	0.08 ± 0.007
**10.5**	3.3 ± 0.31	1.3 ± 0.12	0.06 ± 0.005	**53.4**	3.1 ± 0.29	1.3 ± 0.11	0.07 ± 0.005
**17.5**	5.5 ± 0.59	1.6 ± 0.14	0.05 ± 0.005	**89.0**	5.4 ± 0.51	1.5 ± 0.13	0.06 ± 0.004
